# Scavenging Reactive Oxygen Species Production Normalizes Ferroportin Expression and Ameliorates Cellular and Systemic Iron Disbalances in Hemolytic Mouse Model

**DOI:** 10.1089/ars.2017.7089

**Published:** 2018-08-10

**Authors:** Naveen Kumar Tangudu, Betül Alan, Francesca Vinchi, Katharina Wörle, Dilay Lai, Sabine Vettorazzi, Kerstin Leopold, Maja Vujić Spasić

**Affiliations:** ^1^ Institute of Comparative Molecular Endocrinology, University of Ulm, Ulm, Germany.; ^2^ Molecular Medicine Partnership Unit, Heidelberg, Germany.; ^3^ Institute of Analytical and Bioanalytical Chemistry, University of Ulm, Ulm, Germany.

**Keywords:** heme, oxidative stress, ROS, macrophages, ferroportin, antioxidants

## Abstract

***Aims:*** Release of large amounts of free heme into circulation, overproduction of reactive oxygen species (ROS), and activation of toll-like receptor-4-dependent responses are considered critical for the ability of heme to promote oxidative stress and to initiate proinflammatory responses, posing a serious threat to the body. A deep understanding of the consequences of heme overload on the regulation of cellular and systemic iron homeostasis is, however, still lacking.

***Results:*** The effects of heme on iron metabolism were studied in primary macrophages and in mouse models of acute and chronic hemolysis. We demonstrated that hemolysis was associated with a significant depletion of intracellular iron levels and increased expression of the sole iron exporter protein, ferroportin. The pathophysiological relevance of this mechanism was further demonstrated in sickle cell anemia mice, which, despite chronic hemolysis, maintained high ferroportin expression and increased iron export. We identified a redox active iron species and superoxide as regulators for ferroportin induction by heme. Scavenging the ROS production, by use of a pharmacological antioxidant N-acetylcysteine, prevented ferroportin induction and normalized intracellular iron levels in macrophages and in experimentally induced hemolysis in mice.

***Innovation:*** Our data propose that scavenging ROS levels may be a novel therapeutic strategy to balance intracellular iron levels and systemic iron influx in conditions associated with heme overload.

***Conclusion:*** This study identifies that the pro-oxidant, and not the proinflammatory, actions of heme profoundly impact on iron homeostasis by critically regulating the expression of ferroportin and iron export in hemolytic conditions. *Antioxid. Redox Signal*. 29, 484–499.

## Introduction

During oxidative stress conditions caused by pathological hemolytic diseases and certain bacterial infections, extensive cell damage (hemolysis) occurs. This results in the release of large amounts of hemoglobin and free heme into circulation. Up to 20–280 μ*M* heme can be found in patients suffering from sickle cell disease or thalassemia compared with 0.2 μ*M* heme in healthy volunteers (40, 43). Once released into the blood, free heme and hemoglobin are rapidly scavenged by the plasma proteins hemopexin and haptoglobin, respectively, and cleared from the blood by macrophages and hepatocytes *via* receptor-mediated endocytosis [reviewed in Ref. (7)]. Within the cell, heme is degraded into free (ferrous) iron, carbon monoxide, and biliverdin by heme oxygenase (encoded by the *Hmox-1*) (24, 27, 36).

Innovation Heme, in a concentration range found during hemolytic episodes, increases intracellular reactive oxygen species (ROS) production, which, in turn, signal for the induction of the unique iron exporter ferroportin, subsequently causing iron export from macrophages. These events profoundly affect tightly controlled cellular and systemic iron levels. Given our findings that scavenging ROS production prevented ferroportin induction by heme and normalized iron levels in mice undergoing experimentally induced hemolysis, we envision that pharmacologic antioxidants may be an attractive therapeutic tool to leverage physiological iron levels in conditions wherein heme and ROS levels are high, such as in chronic hemolytic conditions.

Once the capacity of plasma scavenging plasma proteins is overwhelmed, potentially deleterious effects of heme are encountered. These effects are associated with the capacity of heme to readily enter the cell membrane and to impair transmembrane potassium gradient, causing swelling of cells, formation of reactive oxygen species (ROS), and oxidative burst (9, 23, 25, 28, 47).

In particular, the release of iron after heme catabolism and the activation of nicotineamide adenine dinucleotide phosphate (NADPH) oxidase complex have been considered critical for the ability of heme to generate ROS and to promote oxidative stress (2, 5, 7, 28, 38, 42, 49). In addition, as an extracellular signaling damage-associated molecule, heme binds to the pattern recognition toll-like receptor-4 (Tlr4) (18) and promotes sterile inflammation and the activation of the innate immune responses (15, 16, 33, 55), resulting in increased cytokine and lipid mediator production from macrophages (19), enhanced expression of adhesion molecules, and tissue factors on endothelial cells (4, 5, 56). By doing so, heme exacerbates the pathogenesis of hemolytic disorders. Although heme-induced cytokine expression depends on functional Tlr4, ROS production in response to heme is not dependent on Tlr4 signaling (18).

The effects of heme are further potentiated by virtue of its capacity to induce ferroportin, the unique iron-export protein encoded by *Slc40a1* gene (14, 57, 58). High ferroportin levels were measured in macrophages upon heme overload and erythrophagocytosis (12, 13, 31, 32, 37) and in hemolytic murine models of β-thalassemia and phenylhydrazine-induced hemolytic anemia (11, 22, 34).

At the molecular level, induction of ferroportin by heme was shown to be depending on concerted actions between transcriptional regulators Bach1 (Btb and Cnc homology 1) and Nrf2 (nuclear factor erythroid-derived 2-related factor 2), and the binding of the small Maf proteins to Maf recognition elements/antioxidant response elements, located in the promoter region of ferroportin gene (37). Although the transcriptional activation of ferroportin correlated with increase in its protein levels, the most effects of heme on ferroportin protein synthesis were attributed to iron released upon heme catabolism; namely, by using an iron chelator, the increase in ferroportin protein levels by heme was prevented (13, 32). In addition, ferroportin undergoes post-translational control by the systemic iron regulator, hepcidin, whereby binding of hepcidin to ferroportin causes its internalization and degradation, leading to iron retention within the cells (21, 41).

Despite the accumulated evidence showing that heme induced ferroportin expression in heme- and iron-dependent manner in primary macrophages and macrophage cell lines, and the fundamental role of ferroportin in the regulation of iron metabolism, a deep understanding of the consequences of heme overload on cellular and systemic iron responses *in vivo* is, however, still lacking.

In this work, we demonstrate that heme, by its pro-oxidative capability, induces ferroportin and perturbs tightly balanced cellular and systemic iron levels in mouse models of hemolytic conditions. We show that overproduction of ROS by heme underlies ferroportin induction, since scavenging ROS by the use of pharmacological antioxidant N-acetylcysteine prevents ferroportin induction and normalizes iron levels in macrophages and experimentally induced hemolysis in mice. This study reveals the integration between heme and iron metabolism at the level of ferroportin and raises the possibility that limiting the pro-oxidant activities of heme may leverage homeostatic iron responses in hemolytic conditions.

## Results

### Acute and chronic hemolysis in mice promote iron export from the macrophages *via* ferroportin induction

The first objective of this study was to investigate the consequences of heme overload on cellular and systemic iron metabolism in experimental mouse models of acute and chronic heme overload.

**FIG. 1.**
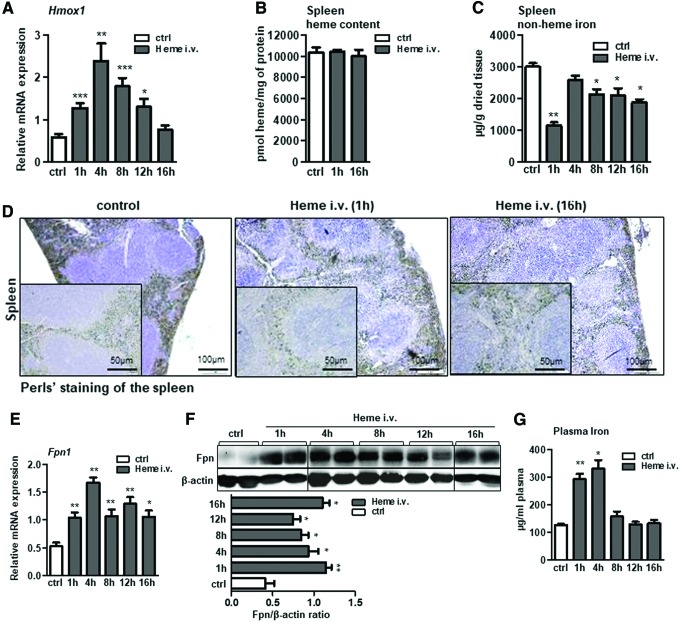
**Acute hemolysis in mice promotes iron export from the spleen** ***via*** **increasing ferroportin expression.** Acute hemolysis was induced in wild-type mice by infusing mice intravenously with heme (i.v. 35 μmol heme/kg body weight) for the indicated period of time. **(A, E)** Relative mRNA expression of *Hmox-1* and ferroportin (*Fpn1*) respectively, measured in the spleen of heme-infused mice by quantitative real-time PCR. **(B, C)** Heme and nonheme iron levels in the spleen, and **(D)** Perls' staining of spleen sections of heme-infused mice; scale bar is 50 and 100 μm. **(F)** Western blot analysis and relative quantification (indicated in histograms) of ferroportin protein levels in the spleen of heme-infused mice. **(G)** Plasma iron levels in heme-infused mice with regard to control mice. Data are presented as mean ± SEM; *n* = 4–5 mice/group; *p-values <0.05, **p-values <0.005, ***p-values <0.0005. Hmox1, heme oxygenase 1; i.v., intravenous; PCR, polymerase chain reaction; SEM, standard error of mean. To see this illustration in color, the reader is referred to the web version of this article at www.liebertpub.com/ars.

Acute hemolysis was induced by infusing mice intravenously with heme (35 μmol heme/kg). Hematological analysis revealed that severe lysis of erythrocytes and decrease in hemoglobin occurred shortly upon heme infusion (Supplementary Table S1; Supplementary Data are available online at www.liebertpub.com/ars), corroborating the concept that excess of free heme enhanced the hemolytic process by impairing erythrocyte integrity (9, 23, 26). We further showed that heme was quickly catabolized in macrophages in a process that associated with significant induction of *Hmox-1* mRNA and with the lack of heme accumulation in the spleen of infused mice (Fig. 1A, B) (53). This suggested that iron, as a product of heme catabolism, may either be stored or exported from the cells.

To address this issue, we measured iron levels in the spleen of infused mice and evaluated the expression of the sole iron exporter protein, ferroportin. Our data revealed that in the conditions of acute heme overload, iron was rapidly exported from the cells, as a significant drop in iron levels in the spleen was detected by the analysis of the nonheme iron content in the spleen of infused mice, and by Perls' staining for iron deposits on spleen sections (Fig. 1C, D). Moreover, we demonstrated that acute heme overload in mice caused for a significant induction in ferroportin mRNA and protein expression, which was maintained at a consistently high level throughout the course of heme infusion (Fig. 1E, F; see also original immunoblot files in Supplementary Data). This effect correlated with a transient elevation of circulating iron levels (Fig. 1G).

**FIG. 2.**
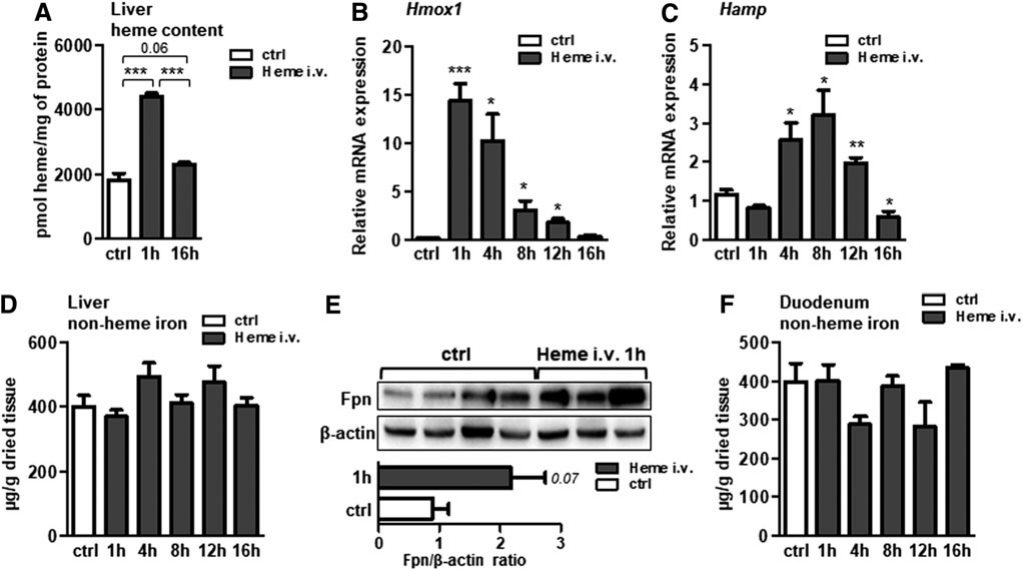
**Acute hemolysis in mice simulates hepcidin and ferroportin induction in liver.** Acute hemolysis was induced in wild-type mice by infusing mice intravenously with heme (i.v. 35 μmol heme/kg body weight) for the indicated period of time. **(A)** Heme levels in the liver of infused mice. **(B, C)** Relative mRNA expression of *Hmox-1* and hepcidin (*Hamp*), respectively, measured in the liver of heme-infused mice by quantitative real-time PCR. **(D, F)** Liver and duodenum nonheme iron content of heme-infused mice. **(E)** Western blot analysis and relative quantification (shown in bars) of ferroportin protein levels in the liver of heme-infused mice. All data are presented as mean ± SEM; *n* = 4–5 mice/group; *p-values <0.05, **p-values <0.005, ***p-values <0.0005.

In contrast to the spleen, the livers of infused mice accumulated heme and significantly increased *Hmox1* expression (Fig. 2A, B), implying that heme was taken up by the hepatocytes and transiently stored (52, 53). The liver responded to heme overload by upregulating the transcription of hepcidin, the iron responsive hormone in a time-dependent manner (Fig. 2C). No obvious iron overload was observed in the livers of heme-infused mice (Fig. 2D), despite elevated plasma iron levels (Fig. 1G) and increased transferrin saturation (59.4 ± 6.8 in infused mice *versus* 34.9 ± 5.8 in control mice, *p* < 0.005). We further measured a more than twofold increase in ferroportin protein expression, however, the data were only marginally under the level of statistical significance (*p* < 0.07) (Fig. 2E). Heme infusion in mice did not affect nonheme iron levels in the duodenum (Fig. 2F).

The consequences of acute heme overload in mice on cellular and systemic iron metabolism, and particularly on modulating ferroportin expression, led us to explore the iron handling capacity of macrophages and ferroportin expression levels under conditions of chronic heme overload. To this end, we used a well-described mouse model of sickle cell disease, HbS mice (44).

**FIG. 3.**
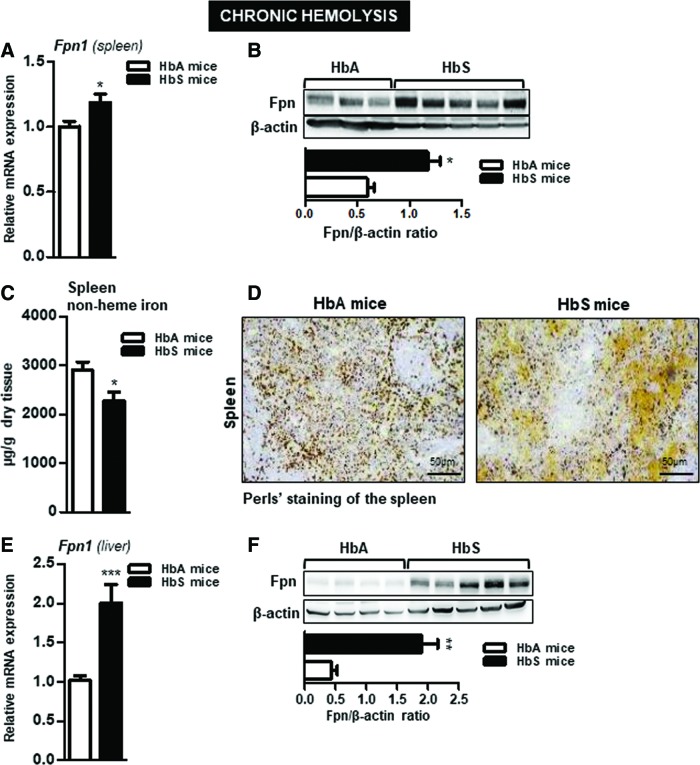
**Chronic hemolysis in mice is coupled with ferroportin induction and iron sparing of macrophages. (A, E)** Relative mRNA expression of ferroportin (*Fpn1*) in the spleen and the liver, respectively, of HbS mice with regard to HbA controls measured by quantitative real-time PCR. **(B, F)** Western blot analysis and relative quantification of ferroportin protein levels indicated by bars, in the spleen and the liver, respectively, of HbS and HbA mice **(C)** The nonheme iron levels in the spleen and **(D)** Perls' stainings of spleen sections (scale bar is 50 μm) of HbS and control HbA mice. Data are presented as mean ± SEM; *n* = 4–5 mice/group; *p-values <0.05, **p-values <0.005, ***p-values <0.0005. HbS mice, sickle cell disease mouse model. To see this illustration in color, the reader is referred to the web version of this article at www.liebertpub.com/ars.

Sustained hemolysis and continuous heme delivery into the tissues of HbS mice associated with increased ferroportin mRNA and protein expression, in the spleen and the liver of HbS mice (Fig. 3A, B, E, F). Similarly to acute heme overload, the spleen of HbS mice showed reduced iron content, demonstrated by Perls' staining for iron depositions and by the analysis of nonheme iron levels in the spleen (Fig. 3C, D). These data suggested that during severe hemolysis, heme-mediated ferroportin induction and low hepcidin in HbS mice (11) served to elevate systemic iron availability, required to sustain high erythropoietic demands in these mice.

**FIG. 4.**
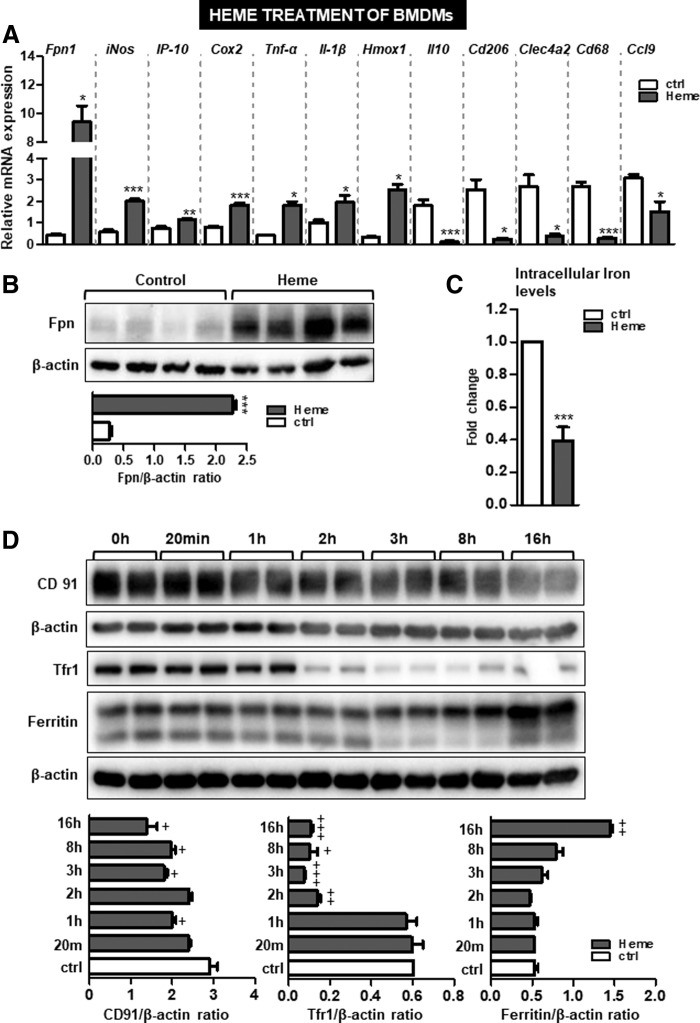
**Excess of free heme alerts macrophage to acquire a proinflammatory phenotype characterized by high ferroportin expression and low intracellular iron content.** Wild-type BMDMs were treated with heme (25 μ*M*; for 16 h) or with vehicle (PBS). **(A)** Relative mRNA expression of ferroportin (*Fpn1*) and of several inflammatory markers was measured by quantitative PCR. **(B)** Western blot analysis of ferroportin protein levels and relative quantification, as shown in bars, was done using ImageJ software. **(C)** Intracellular macrophage iron content measured by total-reflection X-ray fluorescence spectrometer in heme-treated BMDMs. Data represent mean values ± SEM; *n* = 3/group; *p-values <0.05, **p-values <0.005, ***p-values <0.0005. **(D)** Western blot analysis of CD91, TfR1, and ferritin proteins upon treatment of BMDMs with heme for the indicated time. Signals were semiquantified using ImageJ software and shown as bars. +symbol refers to the potential significance between control and heme treatment at different time points, *n* = 2/group. BMDMs, bone marrow-derived macrophages; CD91, cluster of differentiation 91; TfR1, transferrin receptor 1; PBS, phosphate-buffered saline.

Our *in vivo* observations could be recapitulated in isolated macrophages, which upon stimulation with heme (25 μ*M*; 16 h) demonstrated increased ferroportin mRNA and protein expression (Fig. 4A, B) and a significant decrease in the intracellular iron pool (2.2-fold; *p* < 0.01) (Fig. 4C). In a time-line experiment, we showed that heme loading of macrophages decreased the expression of heme–hemopexin complex receptor and transferrin receptor 1 (TfR1) (Fig. 4D), while ferritin levels remained largely unchanged except for an increase in ferritin levels at 16 h post-treatment (Fig. 4D).

### Heme and lipopolysaccharide polarize macrophages toward proinflammatory cells with two opposing iron phenotypes

So far, our results established that in the conditions of acute and chronic heme overload, macrophages acquired high ferroportin expression and an efficient iron export. Consistently, exposure to heme promoted proinflammatory responses in macrophages (Fig. 4A). The observed effects are specific to heme, and possible contamination of heme preparation with lipopolysaccharide (LPS) has been excluded by measuring inflammatory markers after treating macrophages with polymyxin B and heme (Supplementary Fig. S1).

**FIG. 5.**
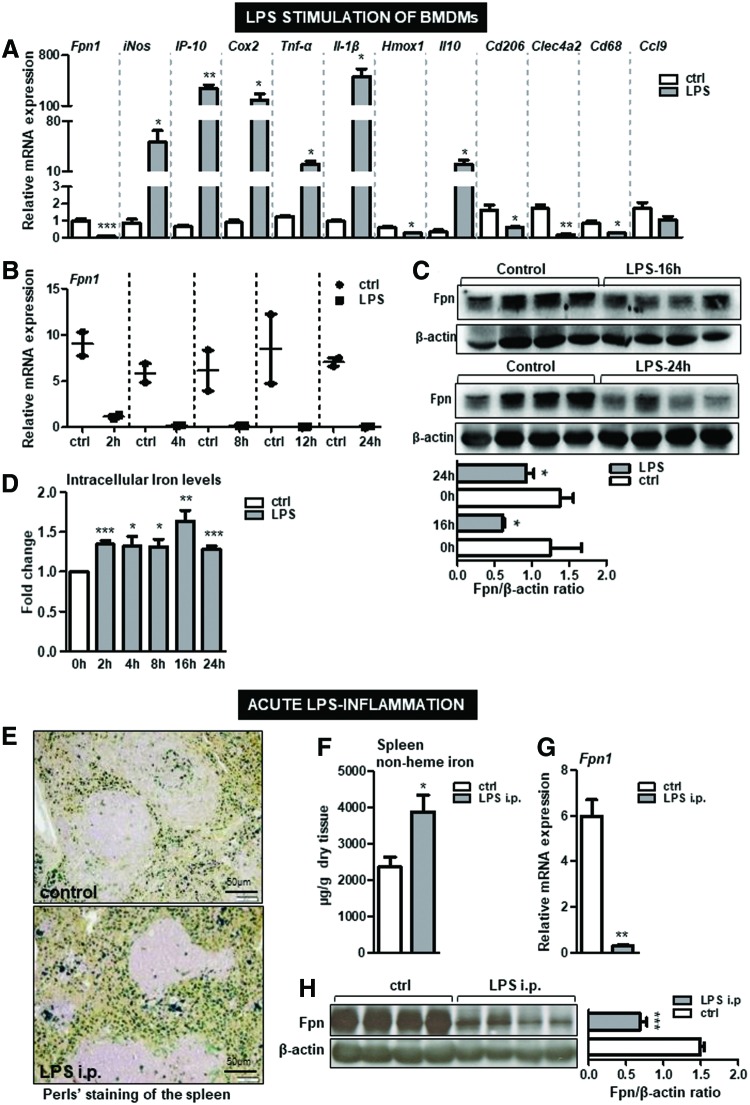
**LPS-triggered proinflammatory macrophages are characterized by low ferroportin expression and enhances iron deposition. (A)** Bone marrow-derived macrophages from wild-type mice were treated with LPS (100 ng/mL; 4 h) or with vehicle (PBS). The mRNA expression of ferroportin (*Fpn1*) and inflammatory markers was measured by quantitative real-time PCR. **(B)** Time-line of ferroportin mRNA expression upon treatment of BMDMs with LPS (100 ng/mL) was measured by quantitative real-time PCR. **(C)** Western blot analysis of ferroportin (Fpn1) protein levels and relative quantification, indicated in histograms, upon treatment of BMDMs with LPS (100 ng/mL) for 16 and 24 h. **(D)** The intracellular iron content upon LPS stimulation of macrophages at indicated time points was measured using total-reflection X-ray fluorescence spectrometer method; *n* = 3–4/group. Wild-type mice were injected with a single sublethal dose of LPS (5 μg LPS; for 6 h). **(E)** The iron deposition was analyzed by Perls' staining of the spleen sections; scale bar is 50 μm **(F)** Nonheme iron content in the spleen of LPS-injected *versus* vehicle-injected mice. **(G, H)** mRNA expression and protein levels of ferroportin were analyzed by quantitative real-time PCR and Western blot analysis, respectively. *n* = 4–6 mice/group. Data are presented as mean ± SEM. *p-values <0.05, **p-values <0.005, ***p-values <0.0005. i.p., intraperitoneal; LPS, lipopolysaccharide. To see this illustration in color, the reader is referred to the web version of this article at www.liebertpub.com/ars.

Importantly, the iron phenotype developed in response to heme overload contrasts the iron responses triggered by LPS, another potent proinflammatory Tlr4 ligand. We showed that simulation of macrophages with LPS resulted in significant reduction in ferroportin mRNA and protein expression and enhanced intracellular iron deposition throughout all time points tested (Fig. 5A–D). Moreover, typical iron retention within the splenic macrophages and significant suppression of ferroportin were measured in mice after LPS injection (Fig. 5E–H). These results indicated that both heme and LPS polarized macrophages toward cells with proinflammatory characteristics, which associated with the development of two distinct iron phenotypes.

**FIG. 6.**
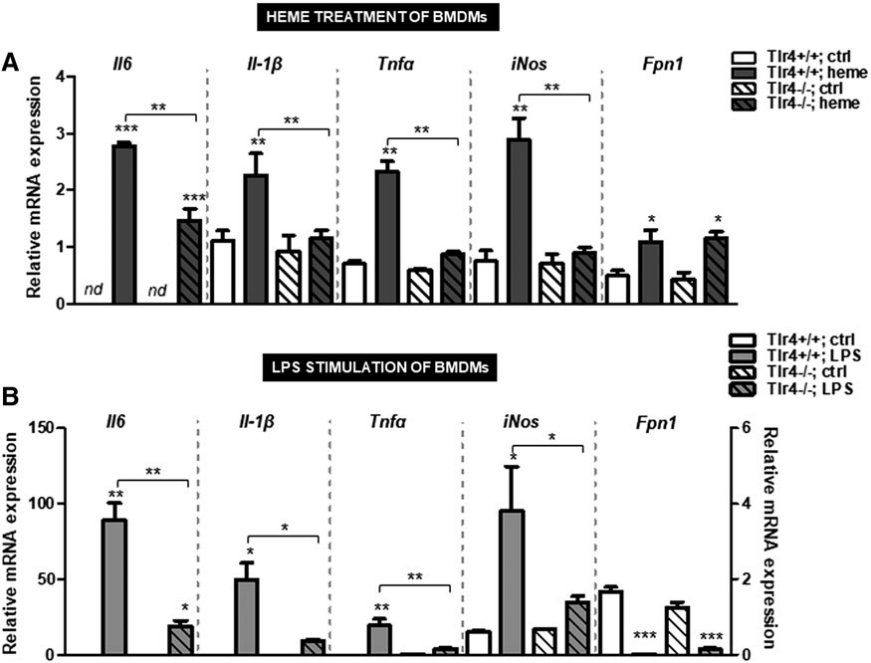
**Ferroportin regulation by heme and LPS does not require the Tlr4. (A, B)** Relative mRNA expression of ferroportin (*Fpn1*) and proinflammatory cytokines in BMDMs from wild-type and *Tlr4*-deficient mice after treatment with heme (25 μ*M* for 16 h) or LPS (100 ng/mL; 4 h). Data were analyzed by quantitative real-time PCR and normalized to the *Gapdh* (*n* = 4). Data represent mean values ± SEM. Statistically significant differences are indicated as *p < 0.05, **p < 0.005, ***p < 0.0005. Gapdh, glyceraldehyde-3-phosphate dehydrogenase; Tlr4, toll-like receptor-4.

We further demonstrated that the proinflammatory Tlr4-dependent activities of both, heme and LPS, were dispensable for ferroportin regulation, since its expression levels were similar between *Tlr4*-deficient and control macrophages (Fig. 6). These results excluded the proinflammatory Tlr4 signaling capacity of heme and LPS, as mediator of ferroportin induction. The expression of proinflammatory cytokines was abolished by the lack of Tlr4 in response to heme and LPS treatment (Fig. 6), supporting previous findings (18, 19).

### Overproduction of ROS after heme overload is critical for ferroportin induction in macrophages

Given that ferroportin induction by heme occurred in a manner independent of Tlr4 (Fig. 6A) and that ROS production in macrophages after heme overload did not require Tlr4 signaling (18, 19), we postulated that increased levels of intracellular ROS might be critical for ferroportin induction. The generation of free radicals by the Fenton reaction and the activation of NADPH oxidase complex have been considered the major form of ROS generated by heme (2, 28, 38). Therefore, the second objective of our study was to explore the potential contribution of ROS and, more precisely, of redox iron and superoxide to ferroportin induction by heme.

The effect of iron, present within the heme moiety, was evaluated by treating macrophages with an iron-free heme analogue (protoporphyrin IX [PPIX]) and upon treatment of cells with heme in the presence of an iron chelator, desferoxamine (DFO).

**FIG. 7.**
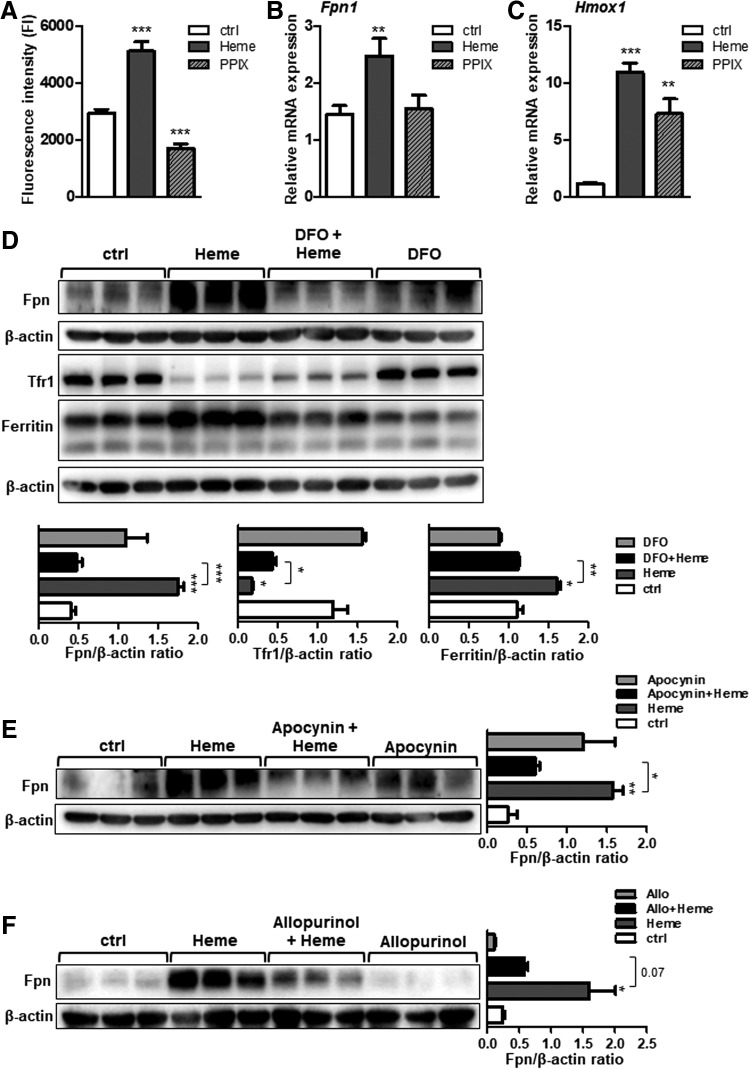
**Iron within heme moiety and the production of superoxide are critical mediators for ferroportin induction by heme in macrophages. (A)** BMDMs were treated with heme (25 μ*M* for 4 h) or with protoporphyrin IX (25 μ*M* for 4 h). The levels of ROS were measured using fluorescence derived from DCFH2-DA dye metabolism and quantified using fluorescence microplate reader. **(B, C)** Relative ferroportin (*Fpn1*) and *Hmox1* mRNA expression were measured using quantitative real-time PCR and normalized to the *Gapdh*. **(D)** Ferroportin, Tfr1, and ferritin protein levels in BMDMs treated with heme (25 μ*M* for 16 h), DFO, (100 μ*M* for 16 h), or in combined treatment with heme and DFO. Signals were quantified using ImageJ software and are shown in bars. **(E, F)** Western blot analysis of ferroportin protein levels in BMDMs treated with heme (25 μ*M* for 16 h), apocynin (100 μ*M* for 16 h), allopurinol (1 m*M* for 16 h), or in combined treatment. Signals were quantified using ImageJ software and shown as bars. *n* = 4–5/group; data are presented as mean ± SEM; *p-values <0.05, **p-values <0.005, ***p-values <0.0005. DFO, desferoxamine; ROS, reactive oxygen species.

We confirmed that the redox active iron, which is derived from heme catabolism in macrophages, is capable of catalyzing ROS formation (Fig. 7A) (19). By contrast, treatment of macrophages with PPIX failed to increase the ROS production and ferroportin expression, implying that iron within the heme moiety was required for the observed effects (Fig. 7B). In contrast, PPIX treatment of macrophages was capable of inducing *Hmox-1* expression (Fig. 7C), indicating that PPIX was taken up by macrophages and catabolized (13). However, the lack of iron after PPIX catabolism reflects the observed lack of ROS production and ferroportin induction. Expectedly, and in line with previous report (13), treatment of macrophages with DFO abolished ferroportin and ferritin induction by heme, whereas TfR1 expression was lowered upon heme and combined treatment with heme and DFO (Fig. 7D).

Collectively, these results underscored the importance of catalytically active iron for enhanced ROS production and subsequent ferroportin induction.

We next explored the potential role of superoxide, which can be produced by several sources such as NADPH oxidase and xanthine oxidase, for ferroportin induction by heme. In our model, the contribution of NADPH oxidase and xanthine oxidase was evaluated by treating the macrophages with inhibitors such as apocynin and allopurinol, respectively. We showed that cotreatment of macrophages with heme and apocynin (Fig. 7E), as well as with heme and allopurinol (Fig. 7F), fully prevented ferroportin induction by heme. These data revealed that inhibiting superoxide at its production site is an effective way to counteract heme-mediated ferroportin induction.

By contrast, in the presence of another ROS species, such as nonradical hydrogen peroxide, ferroportin expression in macrophages was not increased but significantly downregulated (Supplementary Fig. S2). Given the increased ROS levels (which include the production of hydoxyl radicals from hydrogen peroxide and ferrous iron in Fenton chemistry), one may argue that treatment of cells with hydrogen peroxide may cause ferroportin induction. In our experimental setting, we used high concentrations of hydrogen peroxide that may not fully mimic the levels of hydrogen peroxide produced by macrophages after heme overload. Future studies may address how different concentrations of hydrogen peroxide and/or sustained nontoxic hydrogen peroxide levels affect ferroportin expression and whether these effects are present in conditions characterized by heme overload.

Our findings identify redox active iron and superoxide, derived from NADPH oxidase and xanthine oxidase, as sources of ROS critical for ferroportin induction in heme-loaded macrophages.

### Scavenging ROS production after heme overload restores ferroportin expression and normalizes iron levels in the spleen

**FIG. 8.**
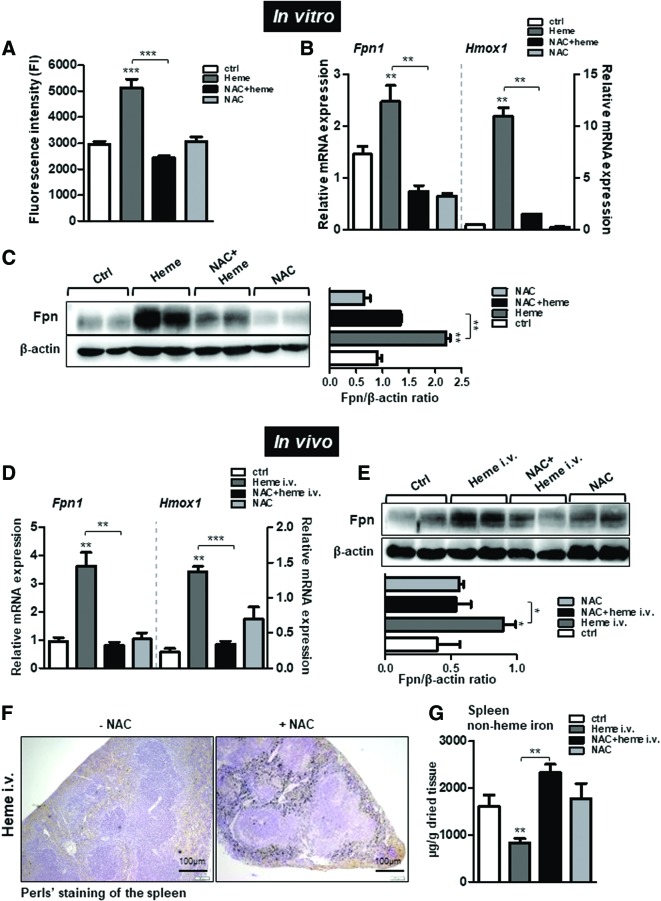
**Scavenging heme-triggered ROS production prevents ferroportin induction and normalizes iron levels in the spleen during experimentally induced hemolysis in mice.** BMDMs were treated with heme, NAC, or in combined treatment with heme and NAC. **(A)** The levels of ROS were measured using fluorescence derived from DCFH2-DA dye metabolism and quantified using fluorescence microplate reader. **(B)** Relative ferroportin (*Fpn1*) and *Hmox1* mRNA expression levels were measured using quantitative real-time PCR and normalized to *Gapdh*. **(C)** Western blot analysis and relative quantification of ferroportin protein levels using ImageJ software in BMDMs treated with heme in the presence or absence of NAC. Acute hemolysis was induced in mice (i.v. 35 μmol heme/kg body weight) for 1 h. Mice received combined treatment of heme and NAC (500 mg/kg NAC 1 h before heme injections) or NAC alone (500 mg/kg; for 1 h). **(D)** Relative mRNA expression of ferroportin (*Fpn1*) and *Hmox-1* in the spleen of control, heme-, NAC, and NAC+heme-injected mice analyzed by quantitative real-time PCR and normalized to *Gapdh.* **(E)** Western blot analysis of ferroportin in the spleen from mice after heme, NAC, or NAC+heme injections. Ferroportin levels were quantified using ImageJ software and band intensity is represented in histogram. **(F)** Perls' staining for iron deposits in the spleen. Scale bar is 100 μm. **(G)** The levels of nonheme iron in the spleen of heme-injected, NAC, or NAC+heme-injected mice; *n* = 4–5 mice/group. Data are presented as mean ± SEM. *p-values <0.05, **p-values <0.005, ***p-values <0.0005. NAC, N-acetyl-L-cysteine. To see this illustration in color, the reader is referred to the web version of this article at www.liebertpub.com/ars.

We next evaluated the effect of antioxidants on ferroportin induction by heme. To this end, we treated macrophages with heme in the presence of N-acetyl-L-cysteine (NAC), a glutathione precursor that acts as a broad antioxidant (Fig. 8A).

We showed that NAC treatment scavenged ROS production and, more importantly, it counteracted ferroportin induction by heme (Fig. 8B, C). In parallel to ferroportin, the expression of *Hmox-1* was increased by heme-triggered ROS production and its induction was prevented by the combined treatment with NAC and heme (Fig. 8B). These data revealed that scavenging ROS where high ROS production already occurred in the system is another promising approach to reducing heme-dependent ferroportin expression.

To validate these *in vitro* observations at systemic levels, wild-type mice were injected with NAC (500 mg/kg, i.p.) 1 h before inducing heme overload (35 μmol heme/kg). We showed that increase in ferroportin mRNA and protein expression by heme was effectively prevented in mice receiving combined treatment of NAC and heme (Fig. 8D, E). Similarly, the induction of *Hmox1* by heme was abolished by cotreatment with NAC and heme, supporting our *in vitro* findings (Fig. 8D). Consequently, iron was retained within the macrophages of the spleen, as demonstrated by the measurement of nonheme iron levels and by Perls' staining for iron deposition (Fig. 8F, G). These results revealed that scavenging ROS production was an efficient way to normalize ferroportin expression and iron levels in mice during acute hemolysis.

## Discussion

Our study highlights the pro-oxidant actions of heme as a critical trigger of cellular and systemic iron disturbances in hemolytic conditions. We show that heme, in a concentration range found during hemolytic episodes, increases intracellular ROS production and consequentially signals for ferroportin induction and subsequent iron export from the macrophages. We propose that this, previously unrecognized, *in vivo* mechanism may represent an important crossroad between heme and iron metabolism, which allows the cell to balance iron deposition and export on the basis of heme uptake.

The finding that heme induces ferroportin is not novel. Previous studies demonstrated that upon exposure of macrophages to heme, or after erythrophagocytosis, ferroportin transcription is upregulated, and its expression on the cell membrane is increased (13, 32). However, those studies were conducted *in vitro* using primary macrophages and macrophage cell lines. To the best of our knowledge, this is the first *in vivo* study addressing the pathophysiology of acute heme overload on cellular and systemic iron levels.

Our results point to an intrinsic macrophage iron circuitry that is established in hemolytic conditions and is maintained even when systemic effects of heme are evoked. For example, in the conditions of pathological heme overload, such as in sickle cell mice, sustained heme delivery into macrophages contributes to continuous transcriptional and translational activation of ferroportin and for an effective iron export from the cells. This mechanism is further boosted by erythropoietic stimuli, which, by suppressing hepcidin expression, potentiate ferroportin stabilization and iron export into the blood (11, 30).

Similar to chronic heme overload conditions, high ferroportin mRNA and protein expression and increased plasma iron and transferrin-bound iron levels were present during acute hemolysis. Under this condition, however, the liver fails to suppress hepcidin expression, reinforcing the view that heme, released as a consequence of intravascular hemolysis, may predominate over hepcidin for the regulation of ferroportin expression. We propose that proinflammatory capacities of heme, rather than increased levels of circulating iron/transferrin-bound iron, signal for hepcidin activation during acute hemolysis. This is supported by the finding that hepcidin induction by heme required Tlr4 signaling (32a) and by the lack of iron deposition in the liver during acute heme overload despite increased plasma/transferrin-bound iron levels (Fig. 2D).

Our data are consistent with the findings that macrophages adopt the characteristics of proinflammatory cells when exposed to an excess of heme (18, 51). Binding of heme to Tlr4 requires the presence of iron within the porphyrin ring of heme and is likely to occur *via* receptor binding site different from that established between Tlr4 and LPS (18). The novelty of our study is the finding that heme and LPS, despite both retaining the ability to bind to Tlr4 and to activate receptor-mediated immune responses, polarize macrophages toward proinflammatory cells with two opposing iron phenotypes. Under heme overload conditions, macrophages acquire an iron phenotype characterized by low intracellular iron and high ferroportin expression. This dramatically contrasts the iron phenotype that develops in response to LPS, hallmarked by high intracellular iron levels and low ferroportin expression (10, 20, 48).

The opposing actions of LPS and heme on ferroportin regulation and iron status might be explained by the differential requirements for iron by the host during inflammation/infections and in hemolytic conditions: by suppressing ferroportin expression during inflammation/infection, macrophages retain iron thereby limiting its availability for pathogen growth. In contrast, heme, as a product of hemolysis and a mediator of sterile and nonsterile inflammation, sensitizes macrophages to acquire a proinflammatory phenotype and to enhance ferroportin expression and iron export into the circulation where iron is required for the erythropoiesis, or eventually, to ameliorate intracellular iron overload and toxicity.

**FIG. 9.**
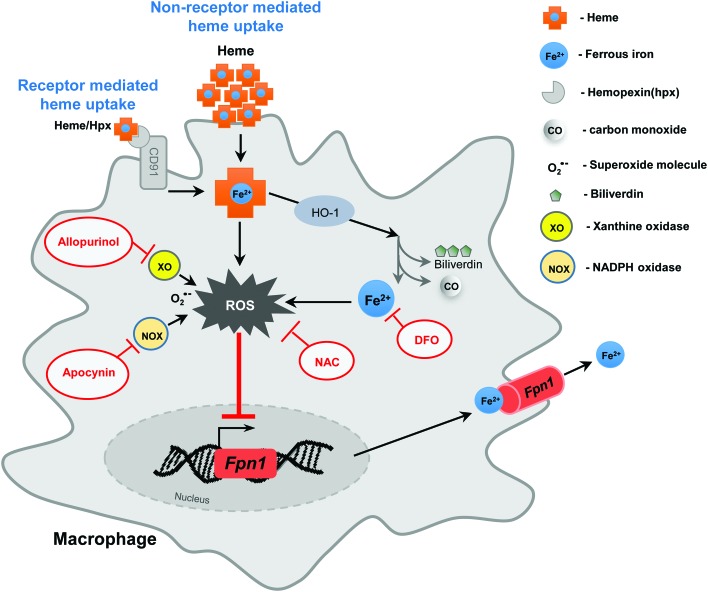
**Orchestration of heme and iron metabolism in macrophages at the level of ferroportin.** The proposed model illustrates the effects of antioxidants and ROS inhibitors on restoring macrophages iron homeostasis during hemolytic conditions. Increased ROS production by heme leads to transcriptional activation of ferroportin (*Fpn1*), increased ferroportin protein levels, and subsequent iron export from the cells. Preventing the release of free iron upon heme catabolism and the production of superoxide from NADPH oxidase and xanthine oxidase sources, by the use of selective inhibitors (such as iron chelator DFO, apocynin, and allopurinol, respectively), abolishes ferroportin induction by heme. In addition, antioxidants, such as NAC, which scavenge ROS production, prevent heme-triggered *Fpn1* mRNA and protein induction and iron egress from the macrophages. NADPH, nicotineamide adenine dinucleotide phosphate. To see this illustration in color, the reader is referred to the web version of this article at www.liebertpub.com/ars.

Further innovative findings of our study are that increased ROS production during heme overload contributes to ferroportin induction and subsequent iron export from macrophages (Fig. 9). For this to occur, iron must be coordinated within the porphyrin ring of heme, since treatment of macrophages with the iron-free heme analogue, protoporphyrin IX, failed to increase both the ROS production and ferroportin expression. Moreover, depleting iron levels by the use of iron chelator DFO prevented ferroportin induction in heme-treated macrophages, re-emphasizing the role of iron for ferroportin induction. This is in line with previous studies, which demonstrated the central role of iron for translational regulation of ferroportin, proposing that this effect is mediated by mechanism that involved iron-responsive protein/iron-responsive elements regulatory network (39). The concept of iron-dependent ferroportin translation in macrophages has, however, been challenged by the generation of mice with selective ablation of Irp2 in macrophages (*Irp2*^LysMCre^ mice), which showed no changes in iron levels and ferroportin expression in macrophages (17). These data reinforce our concept of heme-driven ROS production as a driving force for ferroportin activation, a mechanism that is elicited by macrophages as defense response against iron toxicity and iron-catalyzed oxidative stress (Fig. 9).

Importantly, we demonstrated that treatment with antioxidant NAC, which is an effective scavenger of free radicals, successfully prevented heme-mediated ferroportin induction *in vitro* and in an experimental mouse model of acute heme overload. We believe that these findings could have implications on possible therapeutic strategies in conditions where heme and ROS levels are high. For example, scavenging ROS production by pharmacologic antioxidants in chronic hemolytic conditions may reduce iron efflux into the circulation and lower the generation of unwanted nontransferrin-bound iron fraction; this, in turn, would facilitate the delivery of transferrin-bound iron required for the erythropoiesis.

Antioxidants have already proven efficient in reducing at least some complications associated with sickle cell disease [reviewed in Refs. (8, 29)]. Blocking the pro-oxidant effects of heme was also demonstrated to be beneficial in preventing cell death, tissue damage, and lethality in models of malaria and sepsis, all characterized by heme excess (45). We suspect that rebalancing cellular and systemic iron homeostasis in these conditions might have contributed to the positive effects of antioxidants therapy.

In addition to antioxidants, which neutralize already produced ROS, treatments with drugs that specifically target ROS production at source should be reconsidered. Several enzymes in macrophages are capable of producing ROS themselves. Among those is NADPH oxidase, activation of which results in a burst of superoxide production (1, 3). Previous reports showed that the lack of gp91 phox subunit of NADPH oxidase in mice provided greater protection against oxidative stress during iron overload (49). Another study has shown that free heme induced migration and proliferation of vascular smooth muscle cells, a process dependent on the production of ROS derived from NADPH oxidase activity (38). Our study provides evidence that the increase in cellular oxidant levels, as a result of NADPH oxidase activity, was responsible for ferroportin induction by heme.

We suspect that preventing NADPH oxidase activity rather than, or in addition to, reducing the levels of already produced ROS may constitute a more targeted strategy to leverage cellular and systemic iron levels and reduce pathological consequences of heme/ROS in hemolytic conditions. This idea certainly deserves further investigation. As far as the molecular aspects are concerned, more studies are needed to provide deeper understanding of the mechanism accountable for our observations (Fig. 9).

Collectively, this study advances our current understanding of heme and iron (patho)physiology, identifies ROS species as critical mediators of ferroportin induction during heme overload, and proposes antioxidants as a promising therapeutic approach to restore iron levels in hemolytic conditions.

## Materials and Methods

### Animal experimentations

C57BL/6J wild-type and *Tlr4*-deficient mice, all females, age between 8 and 12 weeks, were maintained on a standard mouse diet containing 200 mg/kg iron (Ssniff, Soest, Germany) under a constant dark–light cycle, and were allowed access to food and water *ad libitum*.

Heme overload in mice was induced by single intravenous (i.v.) injection of freshly prepared hemin (ferric protoporphyrin IX that is reduced to ferrous-protoporphyrin IX, heme, within cells; Sigma Aldrich, St. Louis) at a dosage of 35 μmol/kg of body weight. Hemin was dissolved in 0.1 N of sodium hydroxide (NaOH) and diluted in phosphate-buffered saline (PBS) at a final concentration of 20 mmol/L. pH was adjusted to 7.4 with 0.1 N of hydrochloric acid (HCl). Freshly prepared hemin was injected into the tail vein of mice at a dose of 35 μmol/kg. Control mice were injected with PBS (53). To scavenge the production of ROS, mice were injected intraperitoneally (i.p.) with NAC (500 mg/kg; LKT laboratories, Inc.) 1 h before heme injections. The term “heme” is used generically to refer to both heme and hemin.

Inflammation was induced in mice by single i.p. LPS injection (5 μg LPS; L2630, *Escherichia coli*, serotype 0111:B4, Sigma Aldrich, St. Louis) for 6 h.

Mice were sacrificed by CO_2_ inhalation. Heparinized blood was collected by cardiac puncture and hematological parameters were measured using Scilvet Animal Blood Counter (ABX Diagnostics, Montpellier, France). All animal experiments were approved by and conducted in compliance with the guidelines of the University Ulm Animal Care Committee and the Federal Authorities for Animal Research (Regierungspraesidium Tuebingen, Baden-Wuerttemberg, Germany).

Spleen samples from control HbA and sickle HbS mice (44) have been provided by Professor Tolosano, University of Turin, Italy.

### Preparation of bone marrow-derived macrophages and treatments

Femoral marrows from wild-type mice were flushed and cells were plated at density 1 × 10^6^ cells/mL in culture petri dishes (Becton Dickinson) using Dulbecco's minimal Eagles medium (Invitrogen) supplemented with 10% fetal bovine serum, 10 m*M* sodium pyruvate, 10 m*M* l-glutamine, penicillin, and streptomycin (Sigma Aldrich, St. Louis). The cells were differentiated with 20% mouse L929 fibroblast cell line culture supernatant as a source for macrophage-colony stimulating factor. After 4 days of culture, nonadherent cells were removed and adherent cells, bone marrow-derived macrophages (BMDMs), were washed twice with PBS and the medium was replaced daily for 7 days, when the cells were used for the experiments.

BMDMs were subjected to treatments with the following compounds: heme (25 μ*M*; for 4 and 16 h), heme in combination with NAC (1 m*M*; LKT laboratories, Inc.) 1 h before heme treatment, with PPIX (25 μ*M*, for 4 h; Sigma Aldrich), DFO (100 μ*M*; Sigma Aldrich), allopurinol (1 m*M*; Sigma Aldrich), apocynin (100 μ*M*; Sigma Aldrich), LPS (100 μg/mL, for 4 h; from L2880 *E. coli*, serotype 055:B5; Sigma Aldrich), or with polymyxin B sulfate salt (0.1 μg, 1 μg, and 10 μg/mL) (Sigma Aldrich).

### ROS production

BMDMs were labeled with 2′,7′-dichlorodihydrofluorescein diacetate (2′,7′-DCFH2-DA; Sigma Aldrich) at a concentration of 20 μ*M* for 30 min at 37°C. The mean cellular fluorescence was observed in Leica fluorescence microscope (Leica DMI 6000B, Germany) and measured in fluorescence microplate reader (CLARIO star; BMG Labtech, Germany) at excitation of 488 ± 10 nm and emission of 520 ± 10 nm.

### Measurement of the intracellular iron content using total-reflection X-ray fluorescence spectrometry

Intracellular iron content was measured using total-reflection X-ray fluorescence spectrometry (TXRF) (6). In brief, heme-treated and untreated BMDMs were rinsed with PBS and pelleted in precleaned reaction tubes. To avoid trace contamination, all materials were presoaked in 0.5% nitric acid (HNO_3_) for 12 h, and before use, rinsed three times with ultrapure water (type I water, MilliQ Reference A+; Merck Millipore, Germany). Cell pellets were digested in 1 mL precleaned concentrated HNO_3_ (subboiled; DST-1000 Acid Purification System, Savillex) and briefly vortexed. Ten microliters of digested sample was pipetted on to a precleaned quartz glass carrier in duplicates and dried at 60°C. Each measurement was performed by a *S2 Picofox* benchtop TXRF with a Mo X-ray tube (high-efficiency module; Bruker Nano GmbH, Germany) and a live time of 500 s. A Bayesian deconvolution was applied to the spectra (optimized fit, max. stripping cycles: 100, step width: 1). As an internal standard, 10 μL of Gallium (Ga) solution in 2% HNO_3_ was added to each sample (Gallium standard, 1:10 diluted; AAS Standard 1000 mg/L in 2% HNO_3_, AVS Titrinorm, VWR Prolabo, Belgium). The iron content is measured relative to protein levels in the cells.

### Tissue heme and nonheme iron measurement and Perls' staining

Heme levels in the liver and the spleen were measured using the method described previously (46). In brief, tissues were homogenized in PBS and protein concentration was determined using BCA protein quantification kit from Pierce BCA660 Protein Assay Kit (Thermo Fisher Scientific, Rockford). In duplicates, 0.5 mL of 2 *M* oxalic acid (Sigma Aldrich) and 10 μg of protein were boiled at 95°C for 30 min. The nonboiling samples were used to assess the background fluorescence. All samples were brought to room temperature and centrifuged at 13,500 rpm for 5 min. Emission of fluorescence in samples was determined spectrofluorometrically (CLARIO star; BMG Labtech, Germany) at excitation/emission wave length at 405/662 nm. In parallel, a heme standard curve was run.

The nonheme iron content in the spleen was measured as reported previously (54). Iron deposition was revealed by Perls' Prussian blue staining. Paraffin-embedded spleen tissue sections (of 4 μm) were mounted on frosted slides (VWR, Germany) and stained for iron in the presence or absence of 0.8% 3,3′-diaminobenzidine tetrahydrochloride hydrate (Sigma Aldrich) for 3–5 min.

### RNA isolation, reverse transcription, and real-time polymerase chain reaction

Total RNA was isolated from the spleen or cells using Trizol reagent (Invitrogen) or RNeasy Midi kit (Qiagen, Hilden, Germany) according to the manufacturer's instruction. RNA quality and quantity were controlled using the Nanodrop 2000 system (Thermo Scientific). RevertAid H Minus (M-MulV) reverse transcriptase (RT) (Fermentas), 5 × RT reaction buffer (Fermentas), random primers (200 ng/μl; Invitrogen), and 10 m*M* dNTPs (Bioline, London, United Kingdom) were used to convert 1–2 μg of RNA to cDNA following the manufacturer's instructions. Primers used in the study are listed in Supplementary Table S2.

Quantitative real-time polymerase chain reaction was carried out in 10 μL of reaction volume using SYBR Green I Dye (Invitrogen) on ABI ViiA-7 system (Applied Biosystems). The mRNA abundance of the gene of interest was calculated relative to the expression of the reference glyceraldehyde-3-phosphate dehydrogenase gene *Gapdh* using ΔΔCT method (35).

### Protein isolation and Western blot analysis

Protein extracts were prepared as previously described (54). In brief, flash-frozen spleens or BMDMs were homogenized in RIPA lysis buffer (50 m*M* Tris-Cl pH 8.0/150 m*M* NaCl/1% nonidet P-40/0.5% deoxycholate/0.1% sodium dodecyl sulfate [SDS]) supplemented with the protease inhibitors [Complete Mini (25) ROC 11836153001; Roche Diagnostics, Mannheim, Germany] and phosphatase inhibitors (1 m*M* sodium orthovanadate (Na_3_O_4_Va)/25 m*M* sodium fluoride/1 m*M* phenylmethylsulfonylfluoride; Sigma Aldrich) for 30 min on ice. Cell debris was removed by centrifugation at 13,000 rpm for 15 min at 4°C and protein concentration was determined using the Pierce BCA660 Protein Assay Kit (Thermo Fisher Scientific; Rockford).

Equal amounts of protein extracts were diluted in 5 × Laemmli buffer (0.34 Tris-HCl pH 6.8/0.7% SDS/0.6 *M* dithiothreitol/34% glycerol/bromphenol-blue) and subjected to a 10% SDS-polyacrylamide gel electrophoreses.

After the transfer to nitrocellulose membranes (0.45 μm; BioRad), the membranes were blocked for 1 h in 5% milk powder (Sigma Aldrich) in TBST buffer (20 m*M* Tris-Cl pH 7.5/137.5 m*M* NaCl/0.1% Tween20 (Sigma Aldrich, Germany) and then blotted with antiferroportin (1:500; Alpha Diagnostics Ltd.), antitranferrin receptor 1 (1:500, Tfr1; Zymed laboratories), anti-cluster of differentiation 91 (CD91)/low-density lipoprotein-related protein 1 (1:10,000; Abcam), and antiferritin-H (1:1000 in 2% bovine serum albumin; Cell Signaling Technology) following the manufacturer's instructions. Membranes were washed and incubated with antirabbit horseradish peroxidase-conjugated antibody (1:5000; Invitrogen). Reactions were carried out with Luminata Forte Western horseradish peroxidase substrate kit (Millipore). As loading control, anti-β-actin (1:10,000; Sigma Aldrich) was used in Tris-buffered saline/Tween-20 for 1 h after which membranes were washed and further incubated with secondary antimouse polyclonal antibody (1:10,000; Invitrogen) for 1 h at room temperature. Membranes were washed before the addition of substrate and observed in chemiluminescence detector (BioRad).

The signals were quantified by scanning densitometry and computer-assisted image analysis.^*^

### Statistical analyses

Data were analyzed using GraphPad Prism software and results are shown as mean ± standard error of mean. For the statistical analysis, a one- and two-way analysis of variance was applied, respectively, for the comparison of multiple groups. For pairwise comparisons, the Student's *t*-test (two-tailed; unequal variance) was used. A probability value *p* < 0.05 was deemed statistically significant.

## Supplementary Material

Supplemental data

## Abbreviations Used

BMDMs: bone marrow-derived macrophages
CD91: cluster of differentiation 91
DFO: desferoxamine
Fpn1: ferroportin1
Gapdh: glyceraldehyde-3-phosphate dehydrogenase
HbS mice: sickle cell disease mouse model
HCl: hydrochloric acid
Hmox1: heme oxygenase 1
i.p.: intraperitoneal
i.v.: intravenous
LPS: lipopolysaccharide
LRP1: low-density lipoprotein-related protein 1
NAC: N-acetyl-L-cysteine
NADPH: nicotineamide adenine dinucleotide phosphate
PBS: phosphate-buffered saline
PPIX: protoporphyrin IX
ROS: reactive oxygen species
SDS: sodium dodecyl sulfate
SEM: standard error of mean
TfR1: transferrin receptor 1
Tlr4: toll-like receptor-4
TXRF: total-reflection X-ray fluorescence spectrometry

## Notes

^*^ (ImageJ; www://rsb.info.nih.gov/ij).
