# Supplementary material for: Scavenging Reactive Oxygen Species Production Normalizes Ferroportin Expression and Ameliorates Cellular and Systemic Iron Disbalances in Hemolytic Mouse Model
Source: Antioxid Redox Signal. 2018 Aug 10;29(5):484–99. doi: 10.1089/ars.2017.7089 (PMC6034398; doi:10.1089/ars.2017.7089)
Supplement: Supplemental data [file Supp_Data.zip › Supp_Data.pdf]

Supplementary Data

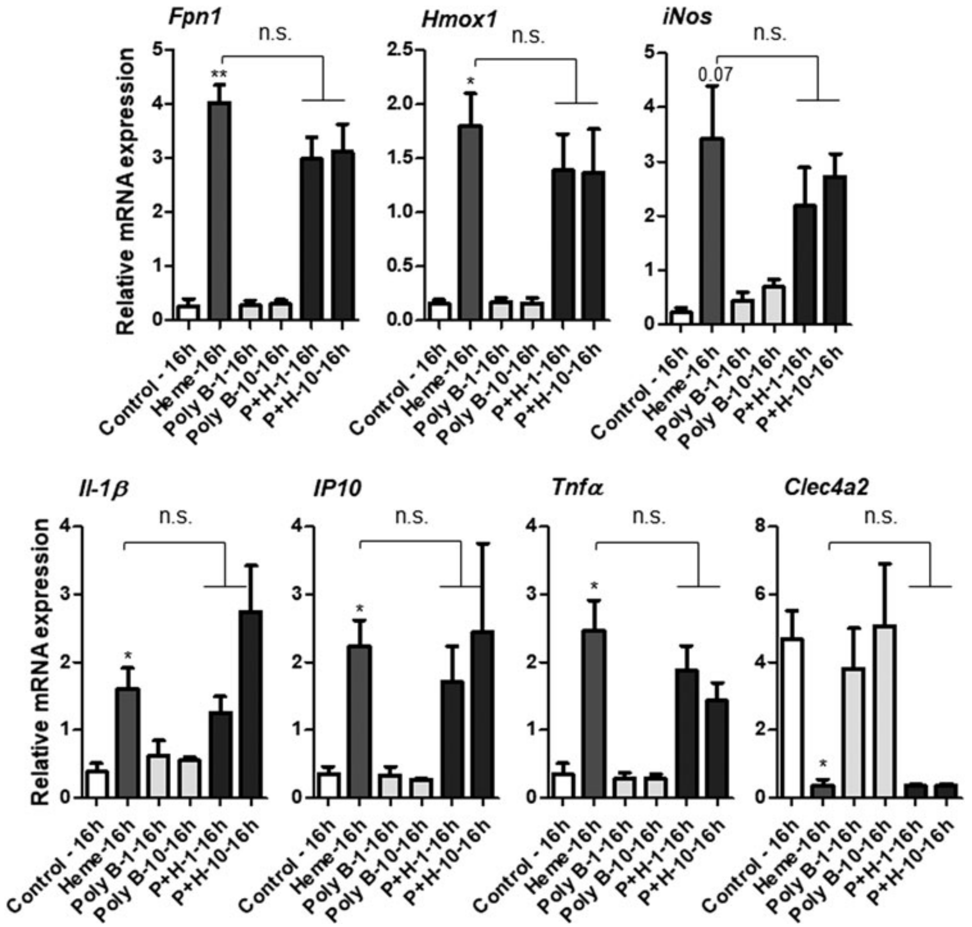

SUPPLEMENTARY FIG. S1. Expression of ferroportin and inflammatory markers in macrophages after treatment with heme in the presence of polymyxin B. Relative mRNA expression of ferroportin (*Fpn1*), *Hmox1*, and inflammatory cytokines (*iNos, Il1b, IP10, Tnfa*, *and Clec4a2*) in bone marrow-derived macrophages from wild-type mice treatment with heme (25 μM for 16 h), polymyxin B sulfate (Poly B; 1 and 10 ng/μl; 16 h), or in combined treatment with polymyxin and heme (P+H). mRNA expression was analyzed by quantitative real-time polymerase chain reaction and normalized to *Gapdh*. ($n = 4$). Data represent mean values ± SEM. Statistically significant differences are indicated as $*p < 0.05$, $**p < 0.005$. Gapdh, glyceraldehyde-3-phosphate dehydrogenase; Hmox1, heme oxygenase 1; SEM, standard error of mean.

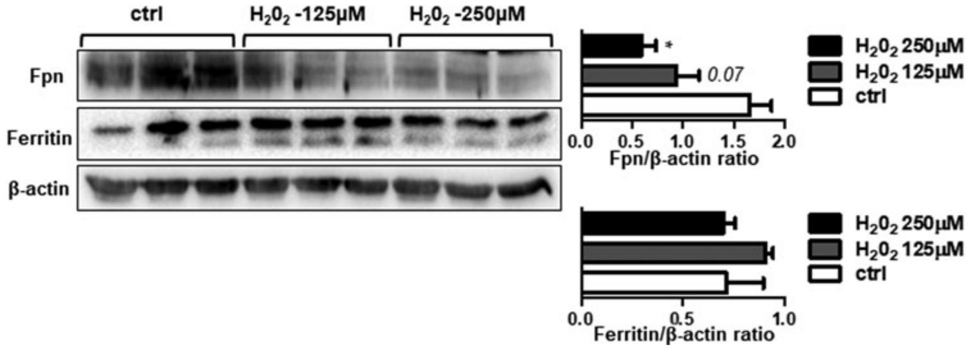

SUPPLEMENTARY FIG. S2. Ferroportin protein levels upon treatment with hydrogen peroxide. Western blot analysis of ferroportin levels in bone marrow-derived macrophages after treatment with hydrogen peroxide (125 and 250 μM; 16 h). Signals were quantified using ImageJ software and shown as bars ($n = 4$). Data represent mean values ± SEM. Statistically significant differences are indicated as $*p < 0.05$.

Supplementary Table S1. Hematological Profile of Mice Upon Acute Hemolysis

	RBC ($10^{6/mm3}$)	*Hg (g/dl)*	*HCT (%)*	*MCV* (μm^3)	*MCH (pg)*	*MCHC (g/dl)*
Control	10.54 ± 0.3	16.3 ± 0.4	55.28 ± 0.9	52.4 ± 1.3	15.48 ± 0.3	29.44 ± 0.22
Heme i.v.	6.94 ± 0.06***	11.55 ± 0.2***	36.25 ± 0.5***	53 ± 0.8	16.98 ± 0.5***	32.1 ± 0.6**

	WBC ($10^{3/mm3}$)	*Lymph (%)*	*Mo (%)*	*Gra (%)*	*#Lym* ($10^{3/mm3}$)	*#MO* ($10^{3/mm3}$)
Control	9.73 ± 0.2	80.4 ± 4.2	3.88 ± 0.5	15.72 ± 3.8	7.85 ± 0.7	0.36 ± 0.1
Heme i.v.	12.53 ± 2.6	54.2 ± 8**	4.7 ± 1.1	41.1 ± 7**	5.3 ± 2.5	0.5 ± 0.3

Hematological parameters were measured from the whole blood of wild-type mice after acute infusion with heme (i.v. 35 μmol/kg heme for 1 h). Data represent mean values ± standard deviation; $n = 4$–5 mice/group. Statistically significant differences were calculated between heme-infused and vehicle-infused mice and are indicated by *p-values <0.05, **p-values <0.005, ***p-values <0.0005. i.v., intraveneous; RBC, red blood cells; Hg, hemoglobin; HCT, hematocrit; MCV, mean corpuscular volume; MCH, mean corpuscular hemoglobin; MCHC, mean corpuscular hemoglobin concentration.

Supplementary Table S2. Primers Used for the Amplification of Target Genes in Real-Time Quantitative Polymerase Chain Reaction

Gene symbol	*Forward sequence (5′-3′)*	*Reverse sequence (5′-3′)*
Fpn1 (Slc40a1)	TGTCAGCCTGCTGTTTGCAGGA	TCTTGCAGCAACTGTGTCACCG
Hmox-1	AGGCTAAGACCGCCTTCCT	TGTGTTCCTCTGTCAGCATCA
Tnfα	TGCCTATGTCTCAGCCTCTTC	GAGGCCATTTGGGAACTTCT
Il6	GCTACCAAACTGGATATAATCAGGA	CCAGGTAGCTATGGTACTCCAGAA
Il1β	GCAACTGTTCCTGAACTCAACT	ATCTTTTGGGGTCCGTCAACT
iNos	GAGACAGGGAAGTCTGAAGCAC	CCAGCAGTAGTTGCTCCTCTTC
IP10	ATCATCCCTGCGAGCCTATCCT	GACCTTTTTTGGCTAAACGCTTTC
Cox2	TCTTTGCCCAGCACTTCAC	ACACCTCTCCACCAATGACC
Il10	TCGGAAATGATCCAGTTTTAC	TCACTCTTCACCTGCTCCAC
Ccl9	TCCAGAGCAGTCTGAAGGCACA	CCGTGAGTTATAGGACAGGCAG
Cd206	GTTCACCTGGAGTGATGGTTCTC	AGGACATGCCAGGGTCACCTTT
Clec4a2	CAAGAGTGAGGAGAACTGCTCC	GCAGCATGAGTGTCCAAGATCC
Cd68	GGCGGTGGAATACAATGTGTCC	AGCAGGTCAAGGTGAACAGCTG
Gapdh	CCCATTCTCGGCCTTGACTGT	GTGGAGATTGTTGCCATCAACGA
