# Supplementary figures and images for: Scavenging Reactive Oxygen Species Production Normalizes Ferroportin Expression and Ameliorates Cellular and Systemic Iron Disbalances in Hemolytic Mouse Model
Source: Antioxid Redox Signal. 2018 Aug 10;29(5):484–99. doi: 10.1089/ars.2017.7089 (PMC6034398; doi:10.1089/ars.2017.7089)

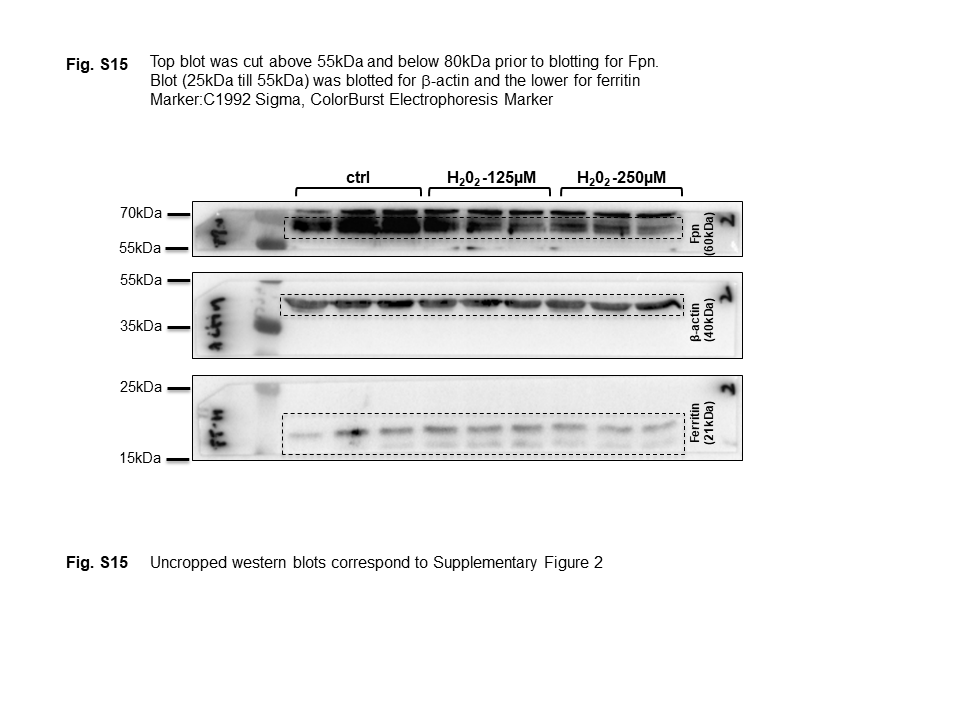

Supplement: Supplemental data [file Supp_Data.zip › Supp_Fig15.tif]

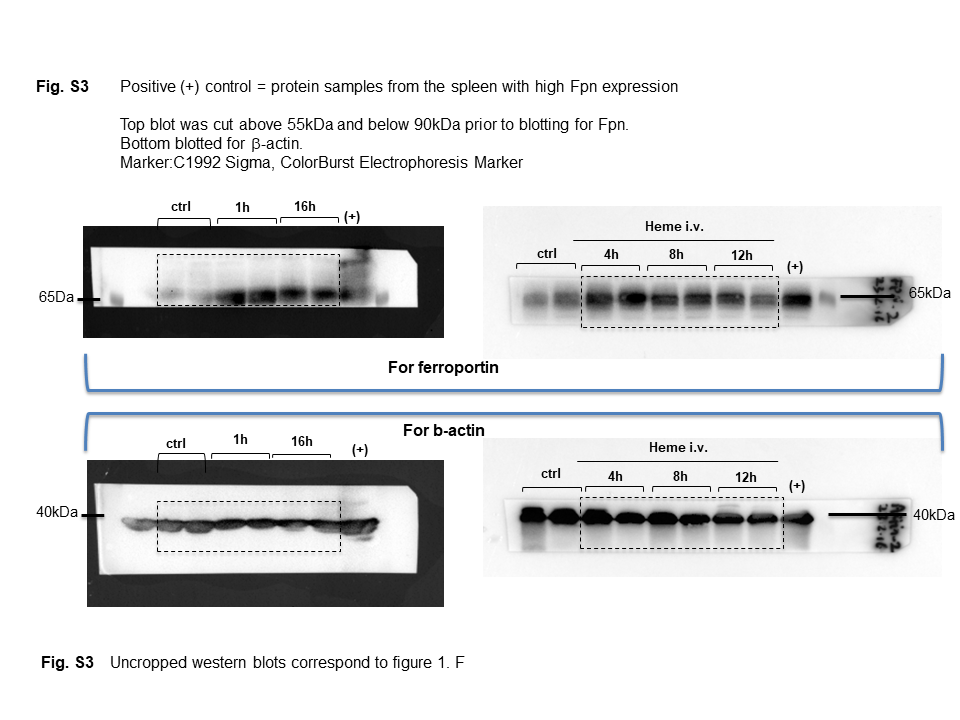

Supplement: Supplemental data [file Supp_Data.zip › Supp_Fig3.tif]

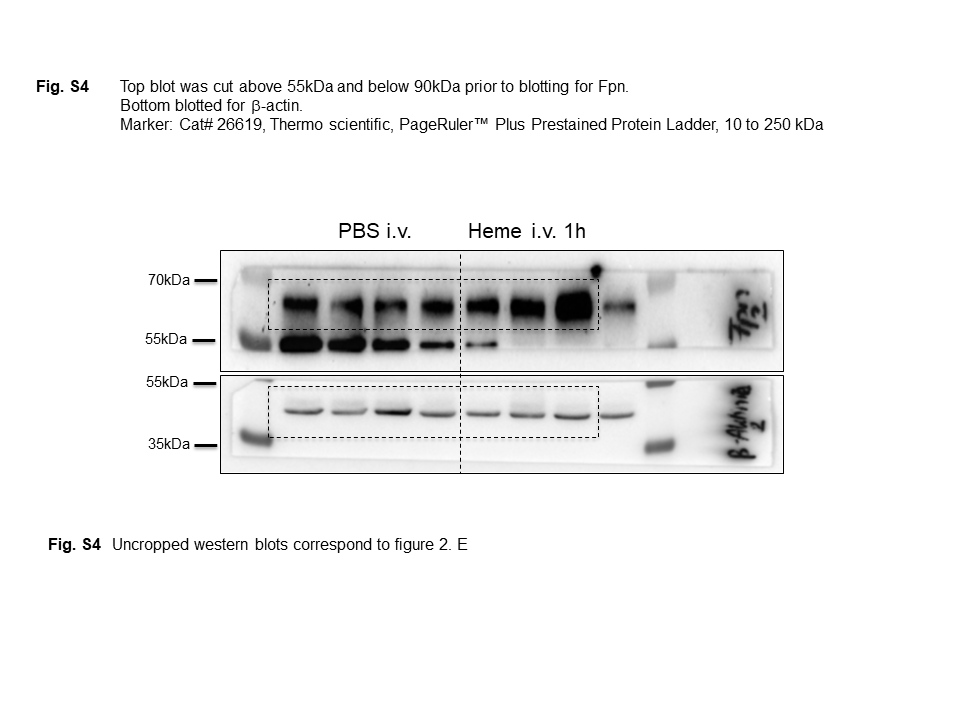

Supplement: Supplemental data [file Supp_Data.zip › Supp_Fig4.tif]

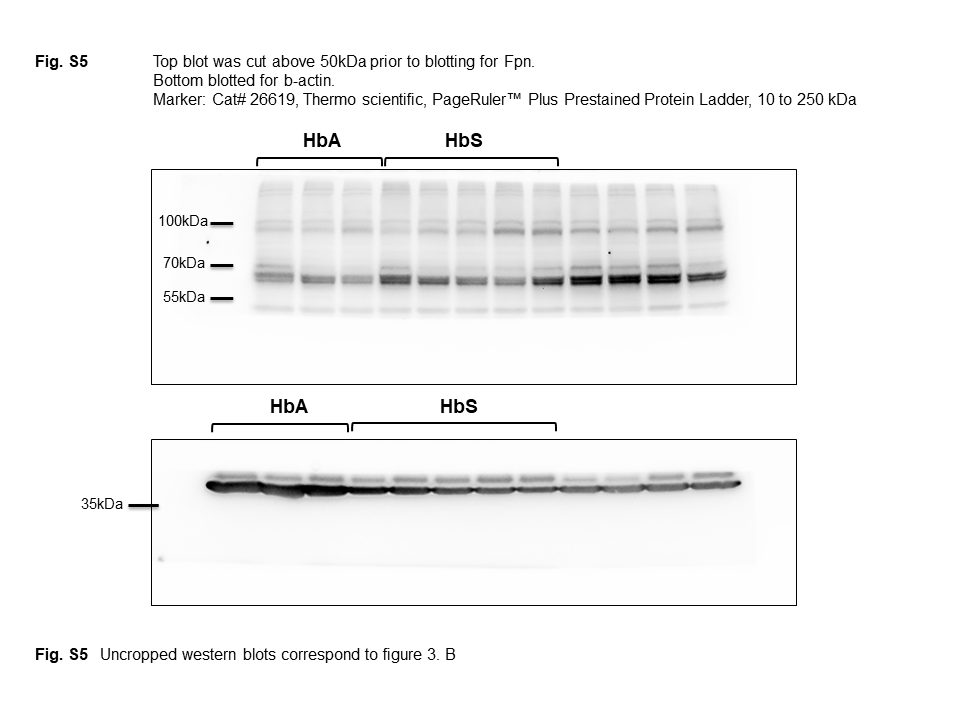

Supplement: Supplemental data [file Supp_Data.zip › Supp_Fig5.tif]

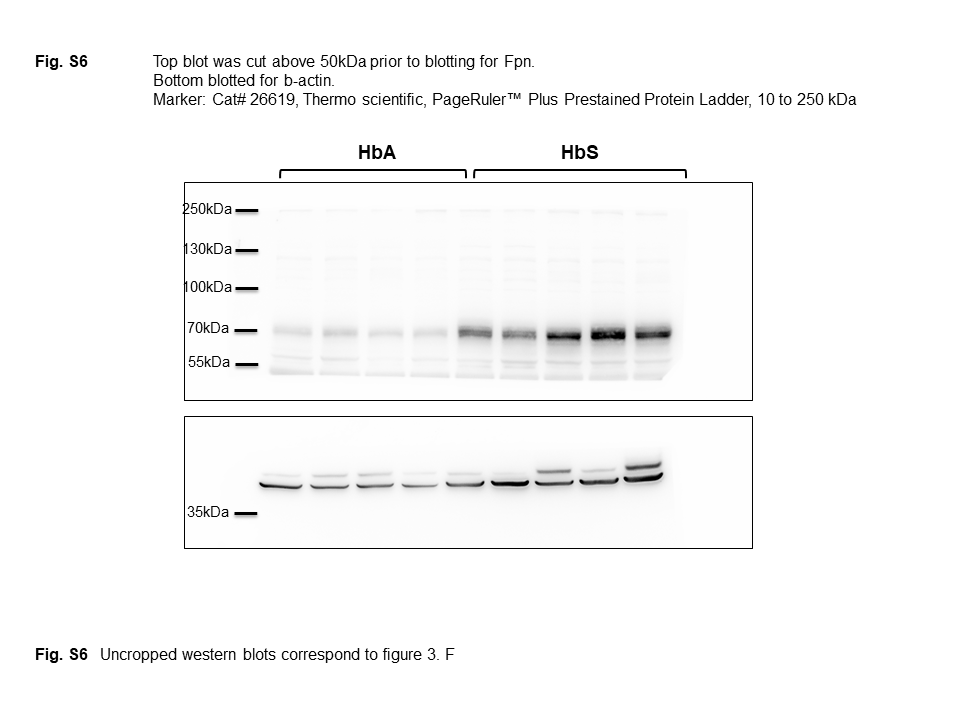

Supplement: Supplemental data [file Supp_Data.zip › Supp_Fig6.tif]

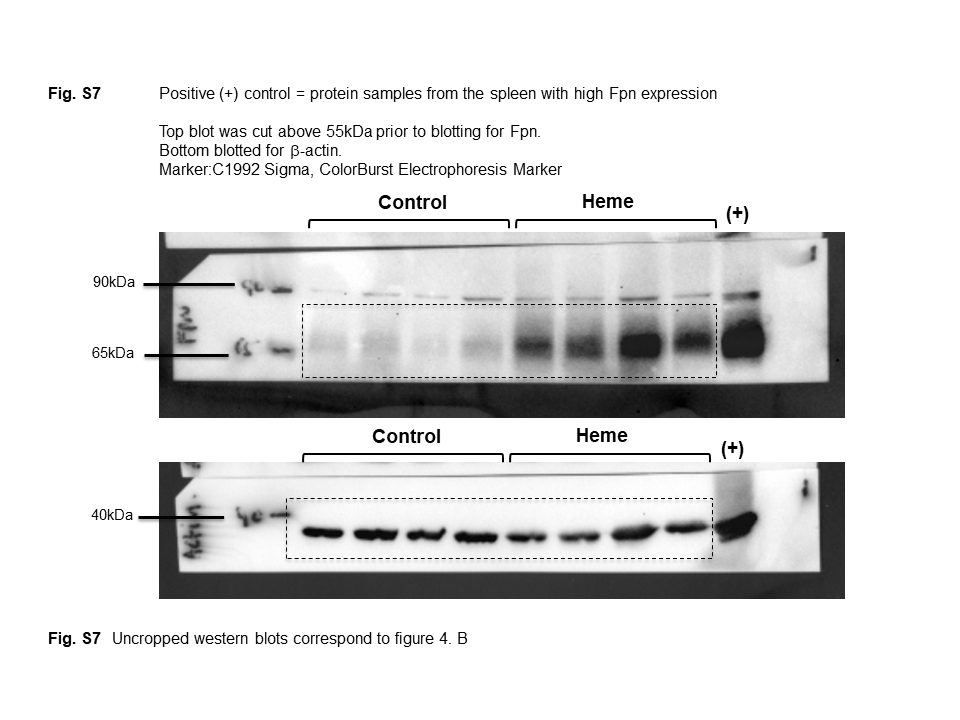

Supplement: Supplemental data [file Supp_Data.zip › Supp_Fig7.tif]

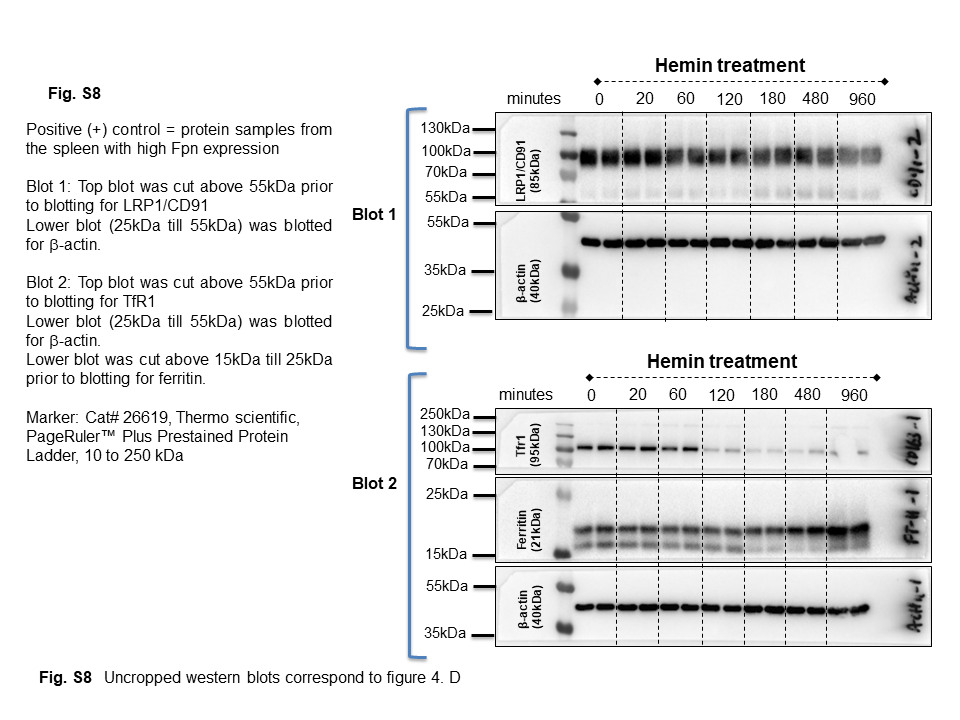

Supplement: Supplemental data [file Supp_Data.zip › Supp_Fig8.tif]

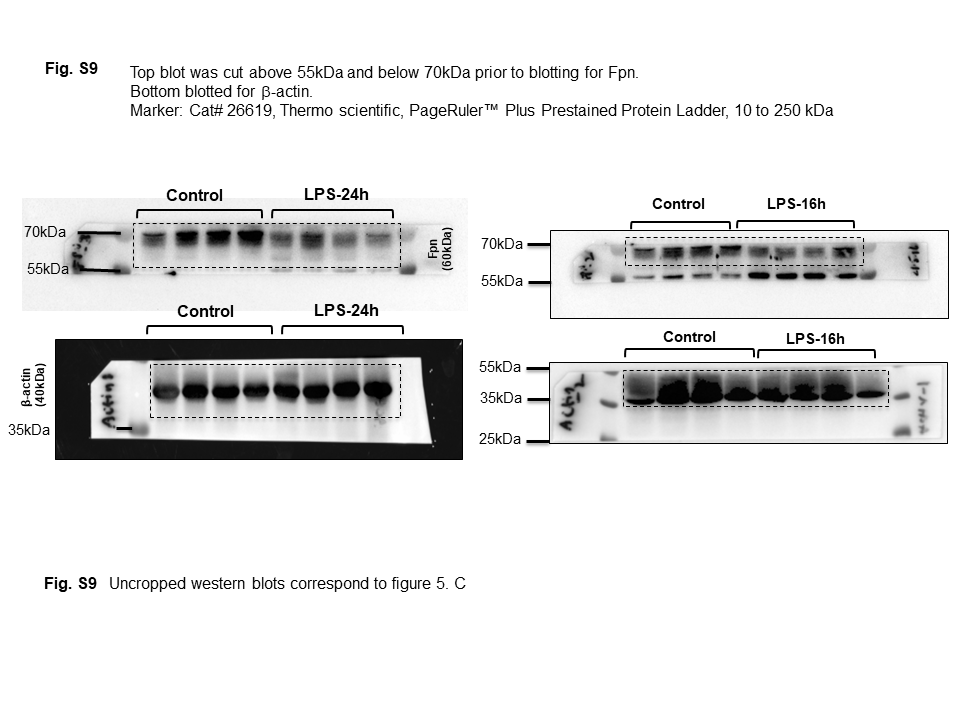

Supplement: Supplemental data [file Supp_Data.zip › Supp_Fig9.tif]

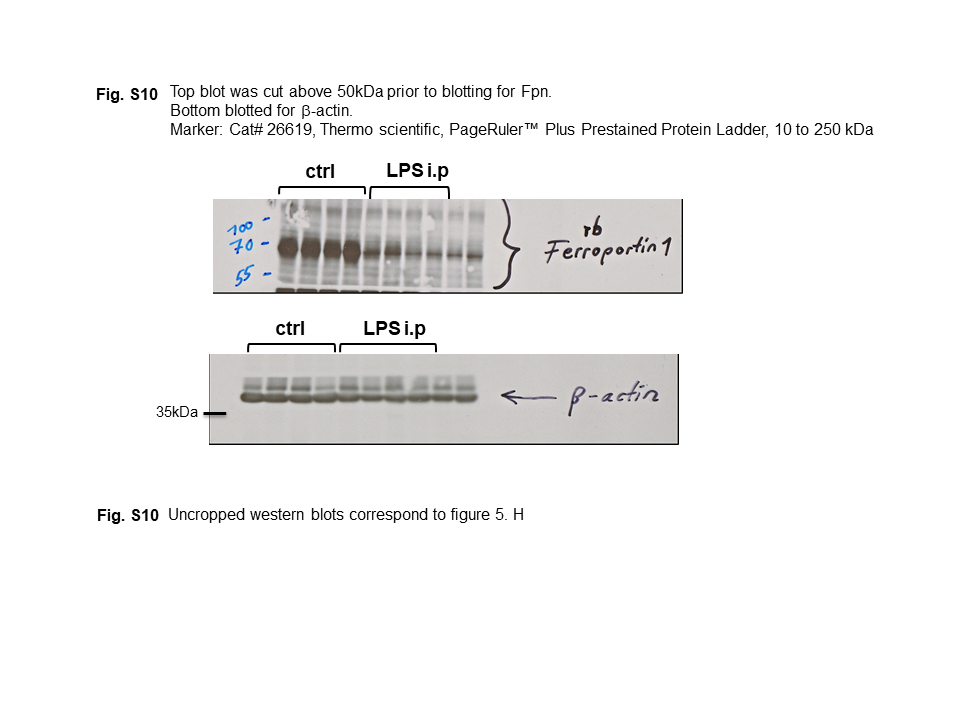

Supplement: Supplemental data [file Supp_Data.zip › Supp_Fig10.tif]

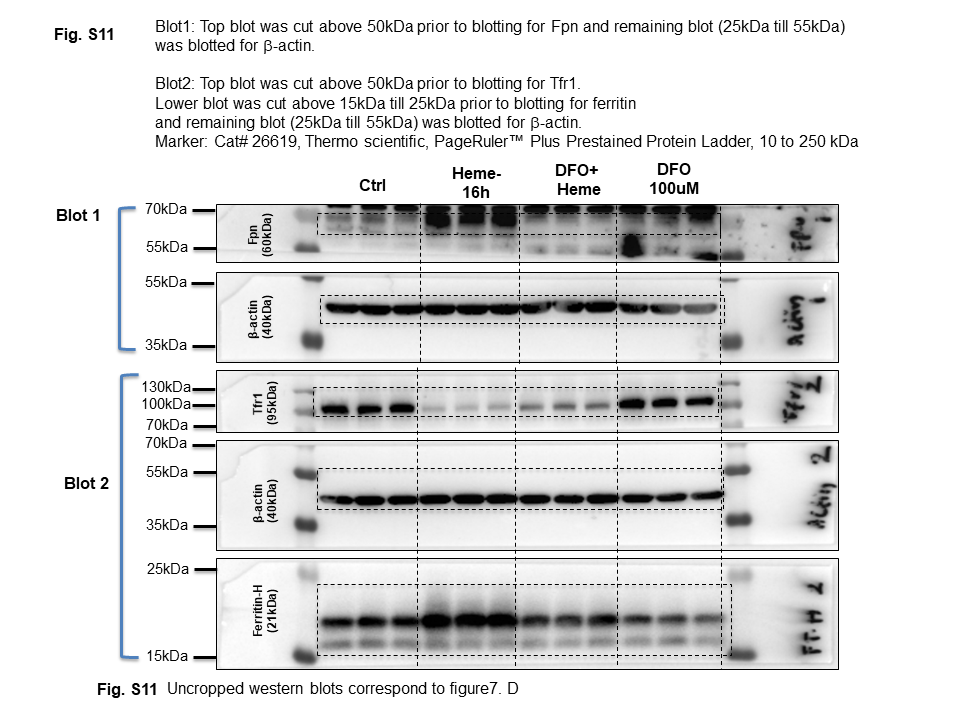

Supplement: Supplemental data [file Supp_Data.zip › Supp_Fig11.tif]

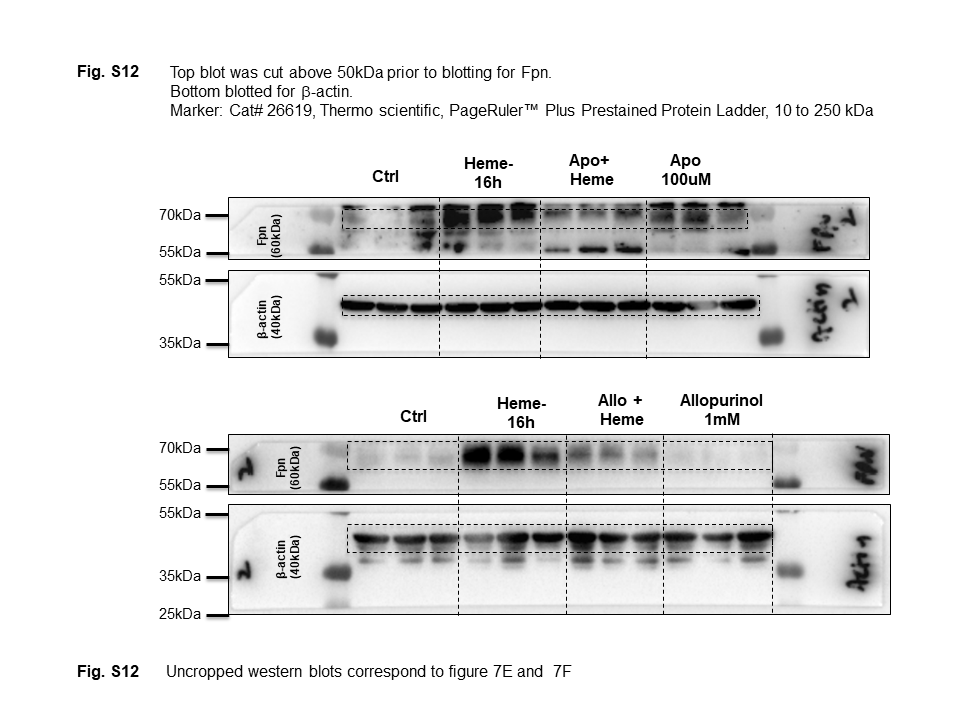

Supplement: Supplemental data [file Supp_Data.zip › Supp_Fig12.tif]

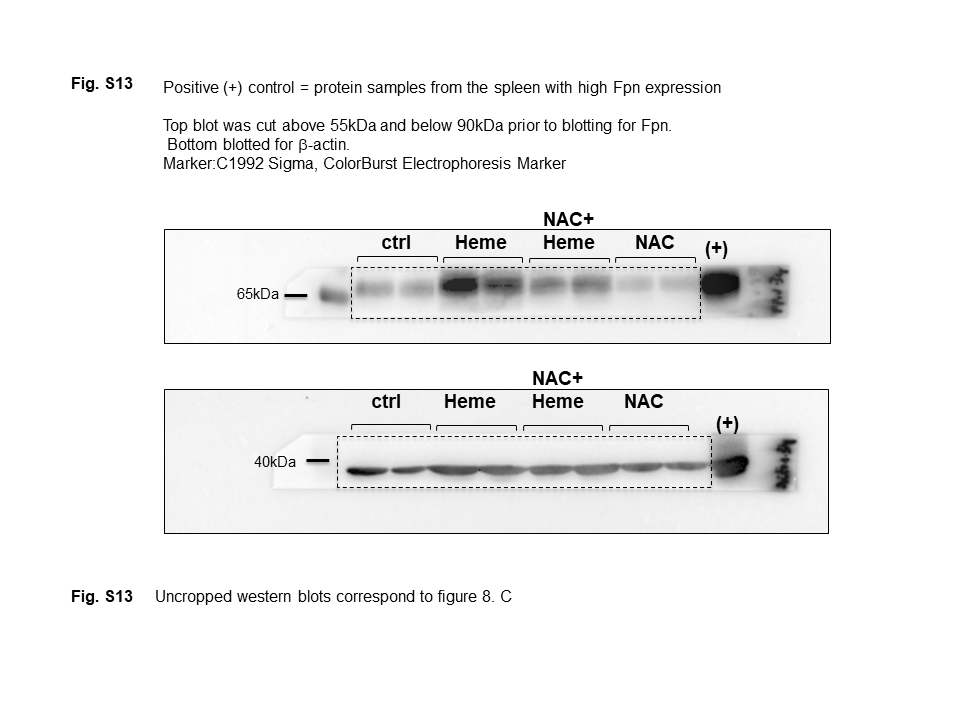

Supplement: Supplemental data [file Supp_Data.zip › Supp_Fig13.tif]

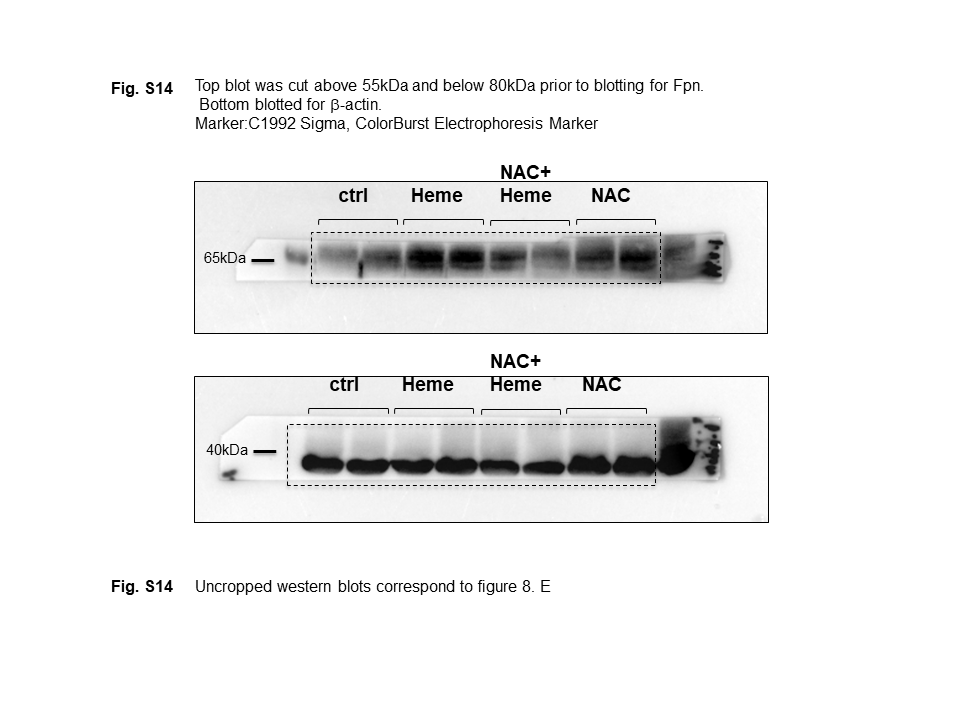

Supplement: Supplemental data [file Supp_Data.zip › Supp_Fig14.tif]
